# Can medial stability be preserved after open wedge high tibial osteotomy?

**DOI:** 10.1186/s43019-020-00071-2

**Published:** 2020-10-01

**Authors:** Hee-June Kim, Ji-Yeon Shin, Hyun-Joo Lee, Kyeong-Hyeon Park, Chul-Hee Jung, Hee-Soo Kyung

**Affiliations:** 1 Department of Orthopaedic Surgery, School of Medicine, Kyungpook National University, Kyungpook National University Hospital, Daegu, South Korea; 2 Department of Preventive Medicine, School of Medicine, Kyungpook National University, Daegu, South Korea

**Keywords:** Pes anserinus, Medial laxity, High tibial osteotomy

## Abstract

**Purpose:**

This study evaluated the medial joint stability after high tibial osteotomy (HTO) releasing the superficial medial collateral ligament (sMCL) without cutting and repairing.

**Methods:**

Twenty-one patients who performed HTO were enrolled. After an L-shaped incision was made in the pes anserinus, the sMCL was released from the distal portion during surgery. After plate fixation, the sMCL was reattached and the pes anserinus was repaired underneath the plate. Plate removal was performed after 31.1 ± 14.2 months. Before HTO, a valgus force of 40 N was exerted at extension for reference values. Before and after plate removal, a valgus force of 40 N was exerted at extension and at a flexion position of 20°. Medial stability was evaluated by measuring the joint line convergence angle (JLCA).

**Results:**

The JLCAs in the extension state before HTO and plate removal were 1.64° ± 1.15° and 1.83° ± 1.36°, respectively; there was no significant difference (*p* = 0.198). There was also no significant difference in JLCA before HTO and after plate removal (*p* = 0.835). There was also no significant difference in JLCA before and after plate removal both at a knee extension and flexion position of 20° (*p* = 0.348 and *p* = 0.456, respectively).

**Conclusions:**

Releasing the sMCL without cutting and repairing the pes anserinus underneath the plate during medial open wedge HTO could facilitate the maintenance of medial joint stability.

## Introduction

High tibial osteotomy (HTO) is a useful surgical option for medial osteoarthritis combined with varus deformity in young active patients [1, 2]. Several surgical options for HTO exist, including the lateral closed wedge and medial open wedge osteotomy [3, 4]. Recently, medial open wedge HTO with locking plates has become favored to avoid co-morbidity associated with fibular osteotomy, which is required for closed wedge osteotomy [5].

During medial open wedge HTO, the medial soft structures should be exposed for the osteotomy site and released for gap opening. Among these structures, the superficial medial collateral ligament (sMCL) is the primary restraint of valgus stress and the pes anserinus also stabilizes the medial side of the knee joint [6, 7]. During HTO, some surgeons prefer subperiosteal elevation and pull-aside without transection. This technique, however, has disadvantages, including narrower view and incomplete correction over 10 mm of osteotomy gap [8]. Other surgeons prefer complete cutting or transection of the sMCL because this provides a good view for surgery and prevents neurovascular damage [8–10]. Additionally, the pes anserinus could be repaired over the plate. After plate removal, severe medial laxity could occur during the conversion of HTO to total knee arthroplasty (TKA), owing to insufficient healing of the sMCL or recutting the pes anserinus over the plate [11].

It is assumed that medial stability could be maintained when the sMCL is released and reattached at the tibial attachment without cutting and the pes anserinus repaired underneath the plate. The aim of the present study was to evaluate medial joint stability before and after plate removal among patients who have undergone HTO with sMCL reattachment and pes anserinus repair under the plate.

## Material and methods

### Patients

This study was approved by the institutional review board (IRB) of the authors’ affiliated institution. This study retrospectively evaluated 21 patients (4 men and 17 women) who underwent medial open wedge HTO for varus deformity and medial osteoarthritis from July 2012 to March 2018. The mean patient age was 56.5 years (age range, 47–61 years). All patients underwent open wedge HTO with locking plates. Tomofix locking plates (Synthes GmbH, Solothurn, Switzerland) were used for five patients and OhtoFix locking plates (Ohtomedical Co. Ltd., Goyang, South Korea) were used for 16 patients.

### Surgical procedure

**Fig. 1:**
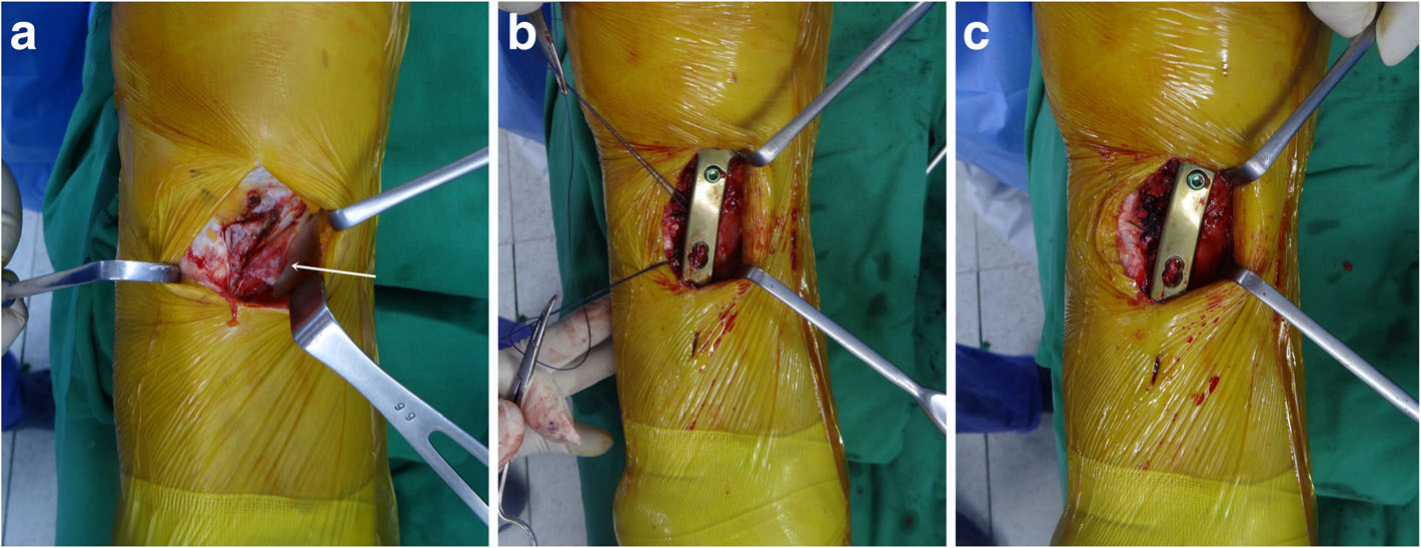
**a** An L-shaped incision of the pes anserinus (*arrow*) was performed to approach the superficial medial collateral ligament and osteotomy site. **b** After the locking plate was fixed, the pes anserinus was passed under the plate using suture thread attached to the pes anserinus. **c** The thread of pes anserinus was sutured to the patellar tendon. Then repair under the plate was performed

All operations were performed by a single surgeon. During HTO, after an L-shaped incision of the pes anserinus was made, the sMCL was released from the distal portion using a periosteal elevator without cutting. Biplane osteotomy was performed and locking plates were used for fixation in addition to one screw for the fourth hole for pes anserinus passage under the plate. The gap was filled with allograft bone chips mixed with autologous bone marrow obtained from the anterior superior iliac spine. Then, the sMCL was reattached, the pes anserinus was repaired underneath the plate, and the final locking screw was fixated (Fig. 1).

### Evaluation methods

**Fig. 2:**
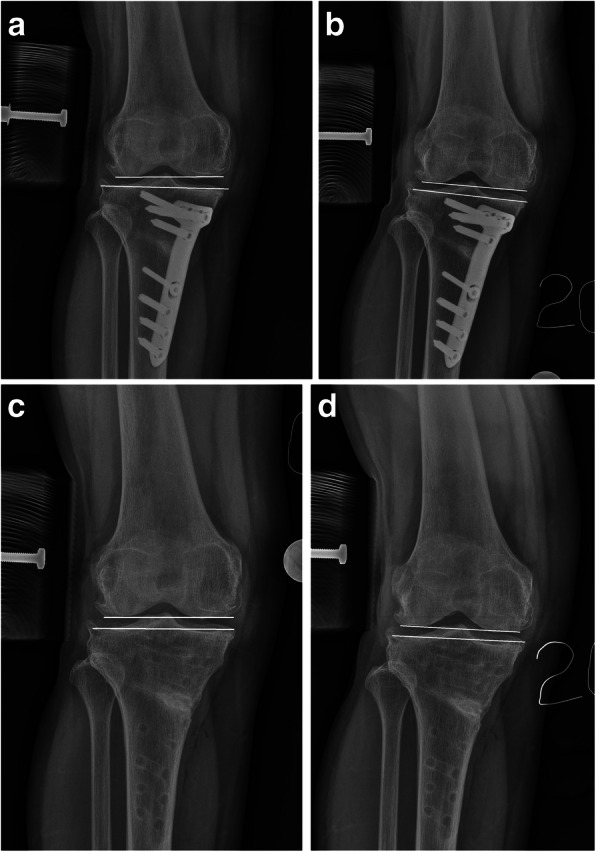
The joint line convergence angle was measured using radiographs. The angle between two lines is joint line convergence angle. **a** A valgus force of 40 N was exerted at extension before plate removal. **b** A valgus force of 40 N was exerted at a flexion position of 20° before plate removal. **c** A valgus force of 40 N was exerted at extension after plate removal. **d** A valgus force of 40 N was exerted at a flexion position of 20° after plate removal

Preoperatively, a valgus force of 40 N was exerted at extension. Medial stability was evaluated by measuring the joint line convergence angle (JLCA). After a mean period of 31.1 months, plate removal was performed and medial stability was also evaluated. Before and after plate removal, a valgus force of 40 N was exerted at extension and at a flexion position of 20°. Medial stability was evaluated and compared by measuring the JLCA (Fig. 2). The change of this angle at each position was compared for evaluating the medial stability. The preoperative JLCA at extension was also compared with the angle before and after plate removal. Clinical scores, including the Hospital for Special Surgery (HSS) score, Knee Society Knee Score (KS), function score (FS), and patellar score was also evaluated before HTO and before plate removal.

### Statistical analyses

The JLCAs at each period were compared using Wilcoxon’s signed-rank test. The clinical results before HTO and before plate removal were also compared using the same method. All statistical analyses were performed using SPSS Statistics version 21.0 (IBM Corp., Armonk, NY, USA). Statistical significance was assumed at a *p* value < 0.05.

## Results

The mean mechanical femorotibial angle was changed from preoperative varus at 7.3° ± 1.7° to postoperative valgus at 3.4° ± 1.5°. The mean HSS score improved from 72.21 to 89.77 (*p* < 0.001). The mean KS and FS also improved from 62.71 to 91.38 (*p* < 0.001) and 61.05 to 89.52 (*p* < 0.001), respectively. The mean patellar score did not change significantly, from 24.62 to 25.00 (*p* = 1.000).

The JLCAs in the extension state before HTO and plate removal were 1.64° ± 1.15° and 1.83° ± 1.36°, respectively; there was no significant difference (*p* = 0.198). There was also no significant difference in JLCA before HTO and after plate removal (*p* = 0.835) (Table 1). There was also no significant difference in JLCA before and after plate removal both at a knee extension and flexion position of 20° (*p* = 0.348 and *p* = 0.456, respectively).

**Table 1:** Joint line convergence angle measurements

	Before HTO operation (°)	Before plate removal (°)	After plate removal (°)	*p* value
At extension	1.64 ± 1.15	1.83 ± 1.35		0.198
	1.64 ± 1.15		1.80 ± 1.25	0.835

*HTO* high tibial osteotomy

## Discussion

This study demonstrated that releasing the sMCL distally without cutting and repairing pes anserinus underneath the plate during medial open wedge HTO could maintain medial joint stability after HTO. It is thought that the released sMCL at the tibial site without transection heals after HTO. Medial stability was also maintained even after plate removal.

Between the two methods of HTO, medial open wedge HTO has the advantage of higher accuracy of correction [3]. Moreover, lateral closed wedge HTO is associated with complications, including peroneal nerve palsy, that can be prevented by performing medial open wedge HTO [12–14]. During medial open wedge HTO, the medial soft structures should be exposed for the osteotomy site and released for gap opening. Among these structures, the sMCL is the primary restraint of valgus stress and the pes anserinus also stabilizes the medial side of the knee joint [6, 7]. However, these structures can be injured during open wedge HTO and this may cause medial instability. For these reasons, several authors have reported that the release of the sMCL for open-wedge HTO should be kept to a minimum to decrease the potential of later valgus instability, because the partial release of sMCL had a similar effect with complete release [15].

A study by Kim et al. [11] demonstrated that medial instability can occur after plate removal during TKA conversion, even if the joint was stable before plate removal. The alignment after HTO was well corrected; however, the medial proximal tibial angle was overcorrected. This could be the result of medial soft-tissue injury during the process of plate removal. When the pes anserinus was repaired over the plate during HTO, pes anserinus dissection over the plate could cause medial instability after plate removal. After this experience, the importance of preserving the medial soft tissue—including by releasing the sMCL distally without cutting and repairing pes anserinus under the plate—became clear.

Cutting the sMCL completely without repair is a good method for approaching the osteotomy site and preventing neurovascular damage [8]; however, this technique could cause medial instability. Pes anserinus repair over the plate is also easier to accomplish than if attempted under the plate. However, soft-tissue irritation might occur and recutting the pes anserinus during plate removal could cause medial instability. Therefore, the present study, wherein the elevated distal portion of the MCL was placed in tissue on grafted bone and the pes anserinus was repaired during open wedge HTO, showed that this is a good method for preventing medial instability.

This study had several limitations, including the small sample size and retrospective study design. Additionally, we did not make comparisons with cases in which the sMCL was cut or in which the pes anserinus was not repaired. Future large, comparative, prospective studies should be performed.

## Conclusions

Releasing the sMCL without cutting and repairing the pes anserinus underneath the plate during medial open wedge HTO could facilitate the maintenance of medial joint stability.

## Data Availability

Not applicable.

## Abbreviations

HTO: High tibial osteotomy
sMCL: Superficial medial collateral ligament
TKA: Total knee arthroplasty
JCLA: Joint line convergence angle
KS: Knee Society knee score
FS: Knee Society function score
IRB: Institutional review board
